# Dexamethasone Treatment of Newborn Rats Decreases Cardiomyocyte Endowment in the Developing Heart through Epigenetic Modifications

**DOI:** 10.1371/journal.pone.0125033

**Published:** 2015-04-29

**Authors:** Maresha S. Gay, Yong Li, Fuxia Xiong, Thant Lin, Lubo Zhang

**Affiliations:** 1 Center for Perinatal Biology, Division of Pharmacology, Department of Basic Sciences, Loma Linda, California, 92350, United States of America; 2 Department of Pediatrics, Loma Linda University School of Medicine, Loma Linda, California, 92350, United States of America; Indiana University School of Medicine, UNITED STATES

## Abstract

The potential adverse effect of synthetic glucocorticoid, dexamethasone therapy on the developing heart remains unknown. The present study investigated the effects of dexamethasone on cardiomyocyte proliferation and binucleation in the developing heart of newborn rats and evaluated DNA methylation as a potential mechanism. Dexamethasone was administered intraperitoneally in a three day tapered dose on postnatal day 1 (P1), 2 and 3 to rat pups in the absence or presence of a glucocorticoid receptor antagonist Ru486, given 30 minutes prior to dexamethasone. Cardiomyocytes from P4, P7 or P14 animals were analyzed for proliferation, binucleation and cell number. Dexamethasone treatment significantly increased the percentage of binucleated cardiomyocytes in the hearts of P4 pups, decreased myocyte proliferation in P4 and P7 pups, reduced cardiomyocyte number and increased the heart to body weight ratio in P14 pups. Ru486 abrogated the effects of dexamethasone. In addition, 5-aza-2'-deoxycytidine (5-AZA) blocked the effects of dexamethasone on binucleation in P4 animals and proliferation at P7, leading to recovered cardiomyocyte number in P14 hearts. 5-AZA alone promoted cardiomyocyte proliferation at P7 and resulted in a higher number of cardiomyocytes in P14 hearts. Dexamethasone significantly decreased cyclin D2, but not p27 expression in P4 hearts. 5-AZA inhibited global DNA methylation and blocked dexamethasone-mediated down-regulation of cyclin D2 in the heart of P4 pups. The findings suggest that dexamethasone acting on glucocorticoid receptors inhibits proliferation and stimulates premature terminal differentiation of cardiomyocytes in the developing heart via increased DNA methylation in a gene specific manner.

## Introduction

The synthesized glucocorticoid dexamethasone is routinely administered to pregnant women at high risk of preterm delivery or to preterm infants to reduce the incidence and severity of respiratory distress syndrome [1,2]. Despite this beneficial effect of dexamethasone treatment, possible long-term adverse effects, including altered cardiovascular and neurological function, have been observed later in life in the exposed individual [3–6]. With regard to the cardiovascular system, negative effects of perinatal glucocorticoid exposure on the development of organs such as the heart and kidney have been noted, including premature death, hypertrophic cardiomyopathy and hypertension [5–8].

Cardiomyocyte terminal differentiation occurs in late fetal development and around birth, and this crucial period of terminal differentiation dictates cardiomyocyte endowment in the heart for life [9]. In humans, cardiomyocytes in term newborn hearts are largely terminal differentiated and the heart grows by enlargement of cardiomyocytes but not proliferation during the postnatal developmental period. In rodents, cardiomyocyte terminal differentiation starts and continues within the first two weeks of postnatal life [10,11], which is an equivalent timeframe in the heart development to the late fetal stage in third trimester of human gestation [12]. Thus, neonatal rats provide a reasonable animal model to study the effect of glucocorticoid treatment on preterm infants at the critical window of the heart development. Glucocorticoids are established cell cycle regulators known to repress the cell cycle, and the glucocorticoid receptor is believed to be the predominant mediators of this effect [13,14]. However, the mechanisms remain largely unknown.

Epigenetic regulation plays an important role during the development. Strong evidence suggests that dynamic DNA methylation orchestrates cardiomyocyte development, maturation and disease [15,16]. It has been shown that dexamethasone has differential effects in the regulation of DNA methylation, which is organ- as well as gene-dependent [17,18]. Thus, the present study sought to determine the effect of dexamethasone treatment in newborn rats on the developing heart and the mechanism of DNA methylation. The hearts were studied at three postnatal development ages of neonatal rats, day 4 (the beginning of binucleation), day 7, and day 14 (the maximum of binucleation). Day 14 represents a mature heart in terms of cardiomyocyte binucleation and terminal differentiation of the heart. The heart to body weight ratio, binucleation, proliferation, cardiomyocyte number, cell size and protein abundance of cyclin D2 and p27 were analyzed.

## Materials and Methods

### Experimental animals

Time-dated pregnant Sprague-Dawley rats were purchased from Charles River Laboratories (Portage, MI). After birth on postnatal day 1 (P1), pups were randomly divided into two groups: 1) saline control, and 2) dexamethasone groups. Newborn rats were treated with tapered doses of dexamethasone (Sigma, 0.5, 0.3, 0.1 mg/kg on P1, P2, and P3 pups, respectively) in the absence or presence of Ru486 (Sigma, 25 mg/kg, P1). Dexamethasone was administered by intraperitoneal injection, and Ru486 was given 30 minutes prior to dexamethasone. Some animals were treated with 5-aza-2'-deoxycytidine (5-AZA, 3 mg/kg) on P1, P2, and P3 through intraperitoneal injection, followed by either saline or dexamethasone (0.5, 0.3, 0.1 mg/kg on P1, P2, and P3, respectively). After treatments, animals were anesthetized using isoflurane and hearts were removed for analyses in P4, P7, or P14 pups. All procedures and protocols in the present study were approved by the Institutional Animal Care and Use Committee of Loma Linda University and followed the guidelines by US National Institutes of Health Guide for the Care and Use of Laboratory Animals.

### Primary cardiomyocyte isolation and culture

Cardiomyocytes were isolated from neonatal rat hearts by enzymatic (0.1% trypsin and 0.5 mg/ml type II collagenase) digestion as previously described [19]. Cells were cultured in Hyclone Medium 199 (Thermo Scientific) containing 10% fetal bovine serum (Gemini Bio-Products) and 1% antibiotics (10,000 I.U./mL penicillin, 10,000 μg/mL streptomycin) at 37°C in 95% air/5% CO_2_.

### Immunocytochemistry

Primary cardiomyocytes were double stained with α-actinin, a cardiomyocyte marker, and Ki-67, a proliferation marker. Cell proliferation was also examined by 5-bromo-2-deoxyuridine (BrdU) incorporation [16]. Briefly, cardiomyocytes isolated from P7 pups were plated on coverslips and allowed 24 hours for attachment. Culture media was then replaced with media containing BrdU (Sigma, 5 μM) for 24 hours. Cardiomyocytes plated on coverslips were fixed with 3.7% paraformaldehyde and permeabilized with 0.5% Triton-X100. The cells were blocked with 1% bovine serum albumin for 1 hour at room temperature before incubation with primary antibodies: mouse anti-α-actinin (Sigma) (1:200), rabbit anti-Ki-67 (Abcam, Cambridge, MA) (1:100), or rabbit anti-BrdU (Abcam) (1:100) at 4°C overnight. Samples were incubated with the secondary antibodies: anti-rabbit AlexaFluor 647 conjugated (1:400; Life Tech.) and anti-mouse AlexaFlour 488 conjugated (1:400; Life Tech.) for 1 hour at room temperature. Nuclei were stained with Hoechst (Sigma) for 1 minute. The immunofluorescence staining was assessed using a Zeiss Axio Imager. All microscope and quantitative analysis was carried out using Image J software.

### Cell number counting

Cardiomyocytes were counted using a hemocytometer. To correct for absolute cell number, cardiomyocyte purity was factored in. Estimates of cardiomyocyte purity were generated using the percent of cardiomyocytes stained with α-actinin from immunocytochemistry results. The hemocytometer values were multiplied by cardiomyocytes fraction calculated using immunocytochemistry, resulting in cardiomyocyte number. This value is expressed as cell number per gram heart weight to account for variations in heart size.

### Western immunoblotting

Hearts of P4 pups were homogenized and protein isolated using the RIPA lysis buffer system (Santa Cruz Biotechnology). Protein concentrations were quantified using the BCA protein assay (ThermoScientific) and all samples were loaded with equal protein onto 10% polyacrylamide gel with 0.1% sodium dodecyl sulfate (SDS). Proteins were then separated by electrophoresis and transferred onto nitrocellulose membranes. Non-specific binding sites were blocked with Tris-buffered saline solution (TBS) containing 5% dry milk. The membranes were incubated with primary antibodies against cyclin D2 (ab3085, Abcam; 1:1000), and p27 (ab7961, Abcam; 1:1000). After washing, membranes were incubated with secondary antibodies. Proteins were visualized with enhanced chemiluminescence reagents and Western blots were exposed to Hyperfilm. To assure equal loading and minimize variability among gels, samples were normalized to GAPDH.

### 5-mC DNA ELISA

DNA methylation in hearts on P4 and P7 pups was determined by measuring 5-methylcytosine (5-mC) using a 5-mC DNA ELISA kit (Zymo Research). The kit features a unique anti-5-mC monoclonal antibody that is both sensitive and specific for 5-mC. The protocol for measurement of 5-mC level is described in the manufacturer’s instruction. Briefly, 100 ng of genomic DNA from hearts and standard controls provided by the kit was denatured and used to coat the plate wells with 5-mC coating buffer. After incubation at 37°C for 1 hour, the wells were washed with 5-mC ELISA buffer and then an antibody mix consisting of anti-5-mC and a secondary antibody was added to each well. The plate was covered with foil and incubated at 37°C for 1 hour. After washed out the antibody mix from the wells with 5-mC ELISA buffer, a HRP developer was added to each well and incubated at room temperature for 1 hour. The absorbance at 405 nm was measured using an ELISA plate reader. The percent 5-mC was calculated using the second-order regression equation of the standard curve that was constructed with negative control and positive controls in the same experiment.

### Statistical analysis

Each experimental group contains a minimum of 4 animals. Data are expressed as mean ± SEM obtained from the number of experimental animals given (*n*). Statistical analysis (p < 0.05) was determined by analysis of variance followed by Neuman-Keuls *post hoc* test or Student's t test, where appropriate.

## Results

### Dexamethasone affects heart development in neonatal rats

**Fig 1:**
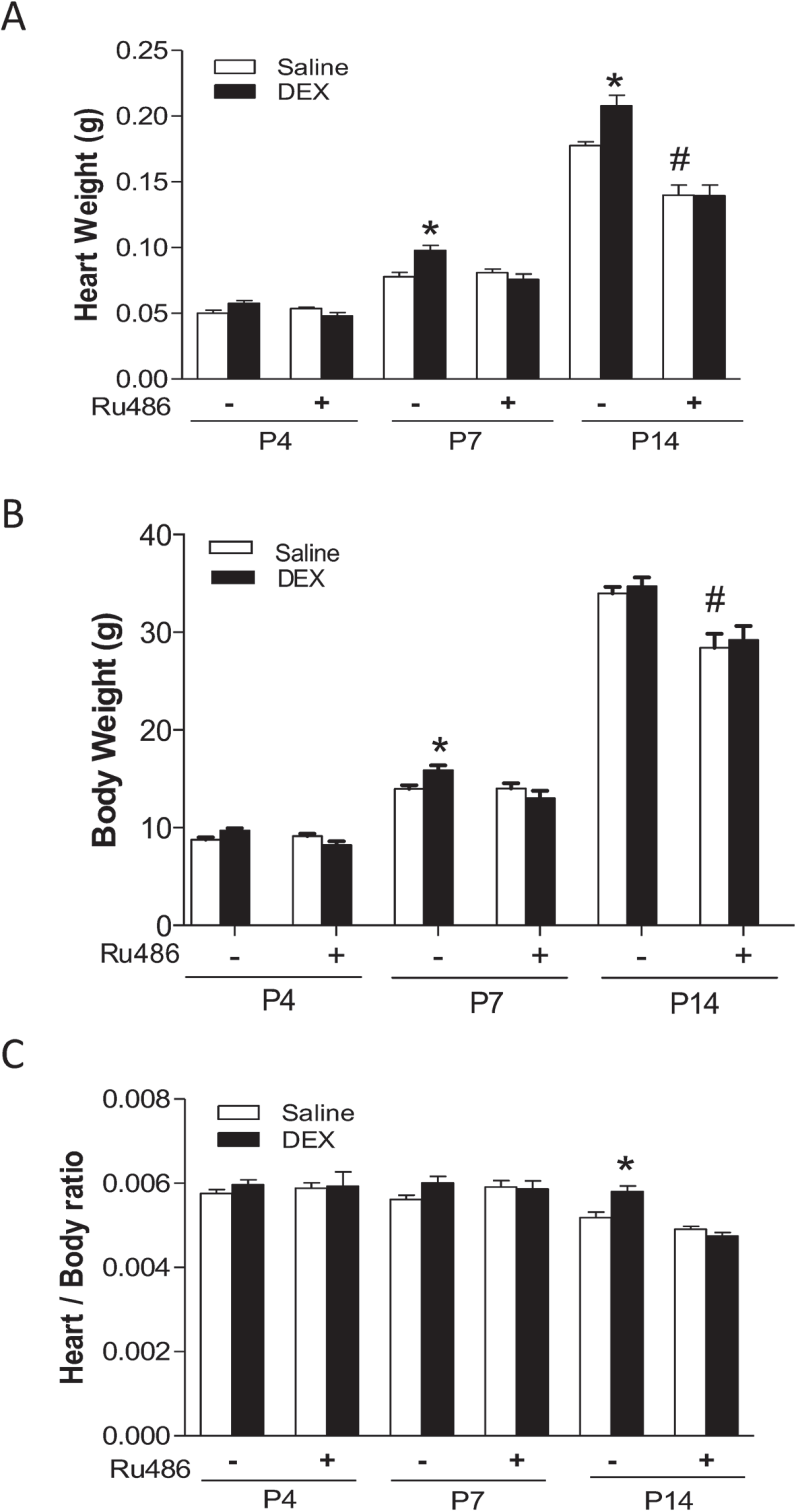
Effect of dexamethasone (DEX) on heart development in neonatal rats. Newborn rats were treated with tapered dose of DEX in the absence or presence of Ru486 during the first three days of postnatal life. Ru486 was administered 30 minutes prior to the DEX treatment. Heart and body weights were determined in day 4 (P4), day 7 (P7) and day 14 (P14) neonatal rats. Data are mean ± SEM, n = 10–21. * p<0.05, DEX *vs*. Saline; # p<0.05, +Ru486 *vs*.-Ru486.

Newborn pups of P1 to P3 were treated with tapered dose of dexamethasone or saline. At P4, P7 and P14, heart weight, body weight and the heart to body weight ratio were evaluated. As shown in Fig 1, in the saline control animals, heart and body weight increased as pups grew, albeit the heart to body weight ratio stayed relatively constant. The dexamethasone treatment increased heart weight in P7 and P14 pups (Fig 1A), but body weight only at P7 pups (Fig 1B), resulting in a significant increase in the heart to body ratio in P14 pups (Fig 1C). To determine the role of glucocorticoid receptor (GR) in the dexamethasone-mediated effects, Ru486 was administered 30 minutes prior to dexamethasone. While Ru486 treatment alone had no significant effects on heart weight, body weight or heart to body weight ratio in P4 and P7 pups, it caused a significant decrease in both heart and body weight in P14 pups (Fig 1). In the presence of Ru486, the effects of dexamethasone were abrogated (Fig 1), suggesting a GR-mediated effect of dexamethasone.

### Dexamethasone induces premature cardiomyocyte binucleation and suppresses proliferation

**Fig 2:**
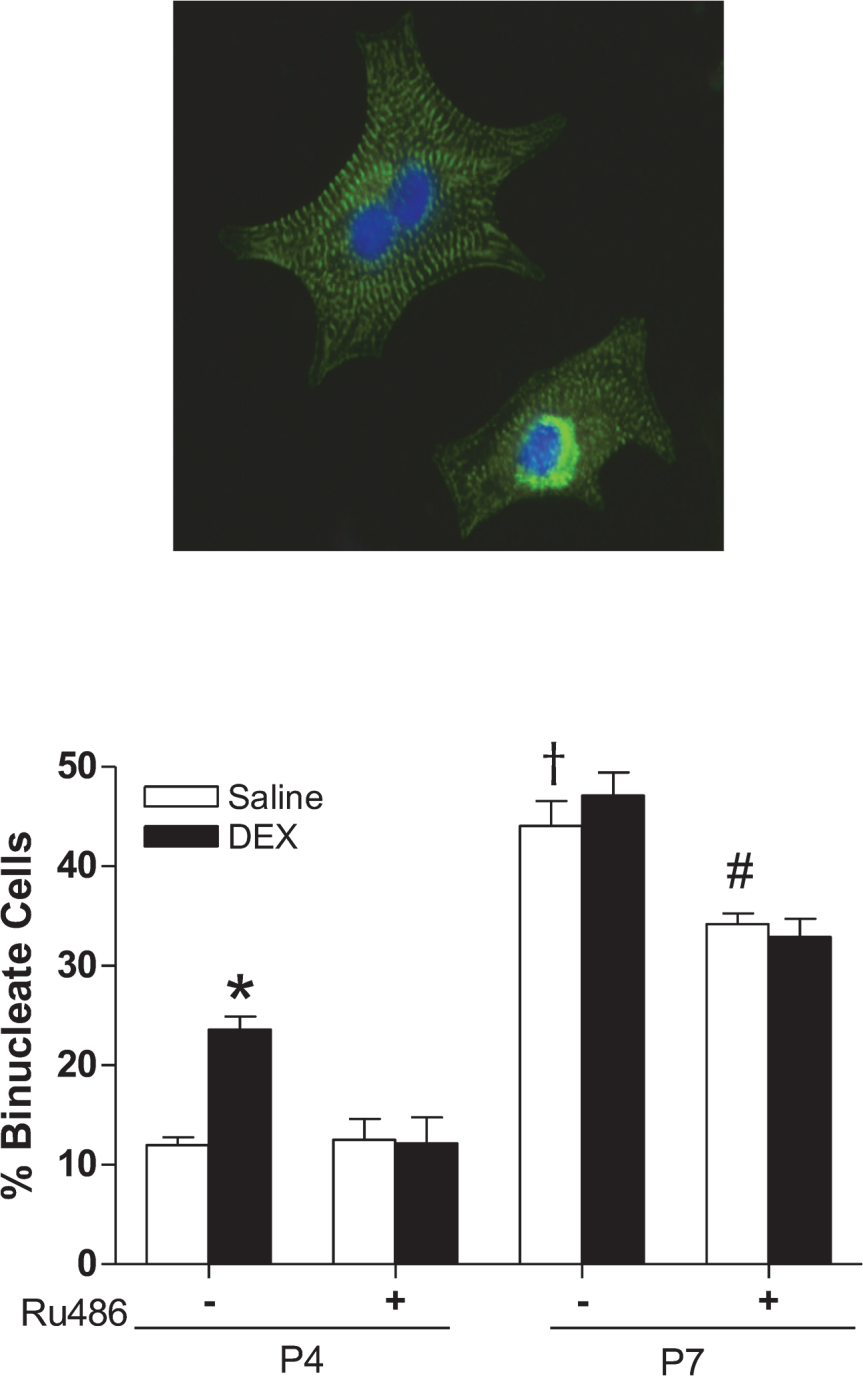
Effect of dexamethasone (DEX) on cardiomyocyte binucleation in neonatal rats. Newborn rats were treated with tapered dose of DEX in the absence or presence of Ru486 during the first three days of postnatal life. Ru486 was administered 30 minutes prior to the DEX treatment. Cardiomyocytes isolated from day 4 (P4) and day 7 (P7) neonatal hearts were stained with α-actinin, and nuclei stained with Hoechst. Representative staining of mononucleated and binucleated cells were shown in the upper panel. Data are mean ± SEM, n = 6–14. * p<0.05, DEX *vs*. Saline; # p<0.05, +Ru486 *vs*.-Ru486; † p<0.05, P7 *vs*. P4.

**Fig 3:**
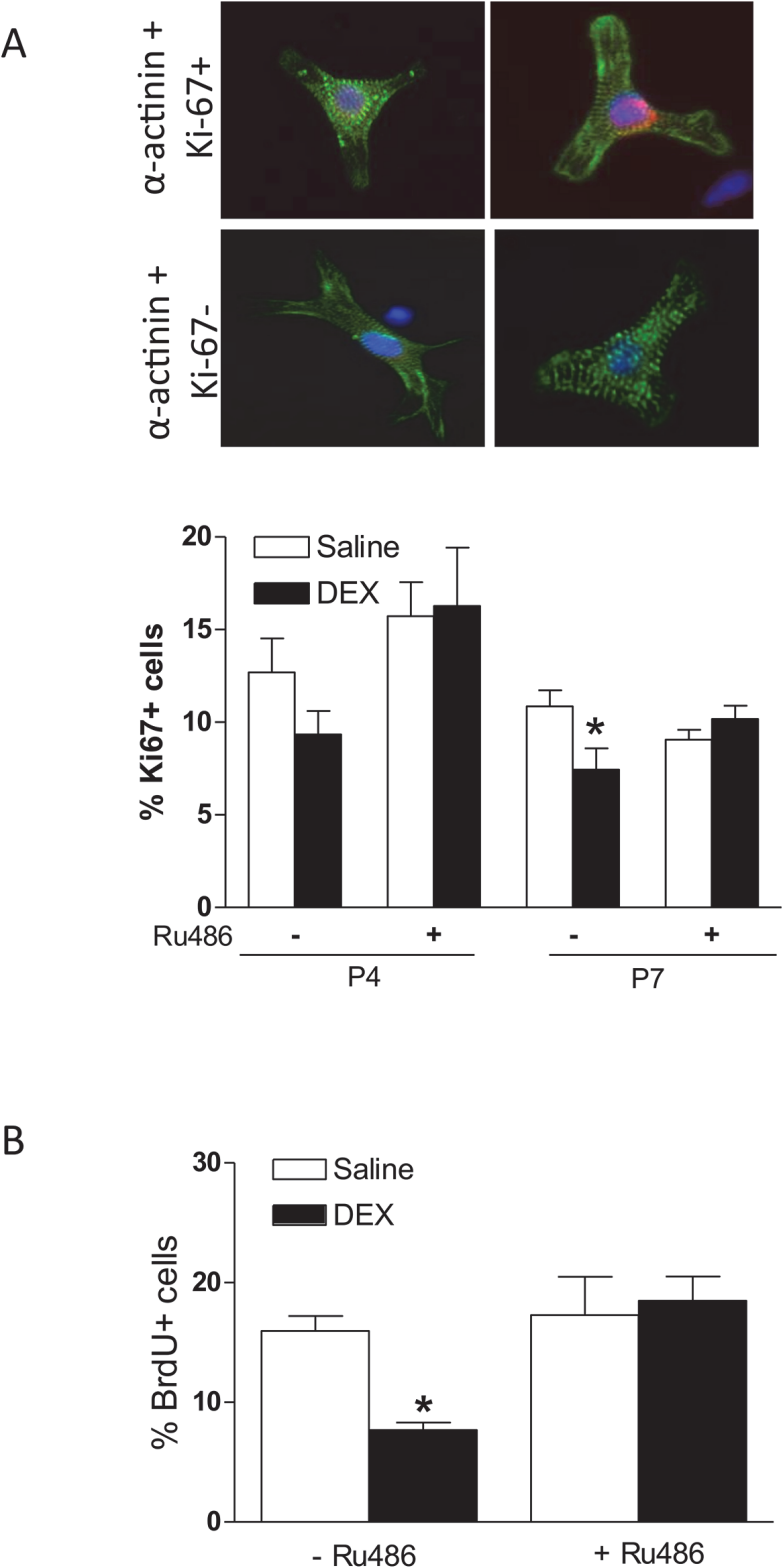
Effect of dexamethasone (DEX) on cardiomyocyte proliferation in neonatal rats. Newborn rats were treated with tapered dose of DEX in the absence or presence of Ru486 during the first three days of postnatal life. Ru486 was administered 30 minutes prior to the DEX treatment. Panel **A**: Cardiomyocytes isolated from day 4 (P4) and day 7 (P7) neonatal hearts were double stained with α-actinin and Ki67, and nuclei were stained with Hoechst. Representative staining of α-actinin and Ki67 co-localization was shown in the upper panel. Panel **B**: Cardiomyocytes isolated from P7 neonatal hearts were examined for BrdU incorporation. Data are mean ± SEM, n = 4–14. * p<0.05, DEX *vs*. Saline.

Binucleation is one of the characteristics of cardiomyocyte maturation and terminal differentiation. Thus, we determined whether dexamethasone treatment influenced cardiomyocyte binucleation in the developing heart. Cardiomyocytes isolated from P4 and P7 pups were stained with a cardiomyocyte marker α-actinin and nuclei were labeled with Hoechst. The percentage of binucleation was scored by counting the number of mononucleated and binucleated cells using microscopy. As shown in Fig 2, in control animals, there was a development-dependent increase in percent binucleated cells in the hearts of P4 and P7 pups. The dexamethasone treatment resulted in a significant increase of percent binucleated cells in the hearts of early developmental age of P4 pups, which was blocked by Ru486 (Fig 2). Ru486 treatment alone, while it had no significant effect on cardiomyocyte binucleation in P4 pups, significantly decreased percent binucleated cells in the hearts of P7 pups (Fig 2). Because binucleation of cardiomyocytes is an early indicator showing the transformation of hyperplasia to hypertrophy growth and suggesting cell destiny from proliferation to differentiation, the proliferation of cardiomyocytes was determined by double staining of α-actinin and Ki67, a marker for cellular proliferation. The dexamethasone treatment showed a tendency in decreasing percentage of Ki67-positive cardiomyocytes in P4 pups, and it significantly decreased Ki67-positive cardiomyocytes in P7 pups (Fig 3A). Ru486 treatment alone had no significant effect on cardiomyocyte proliferation in either P4 or P7 pups, but abrogated the dexamethasone-induced inhibitory effect on myocyte proliferation (Fig 3A). To confirm the proliferation data observed with Ki67, BrdU incorporation was also examined in P7 cardiomyocytes. As shown in Fig 3B, the dexamethasone treatment significantly decreased BrdU incorporation in P7 cardiomyocytes, which was blocked by Ru486.

### Dexamethasone decreases cardiomyocyte endowment

**Fig 4:**
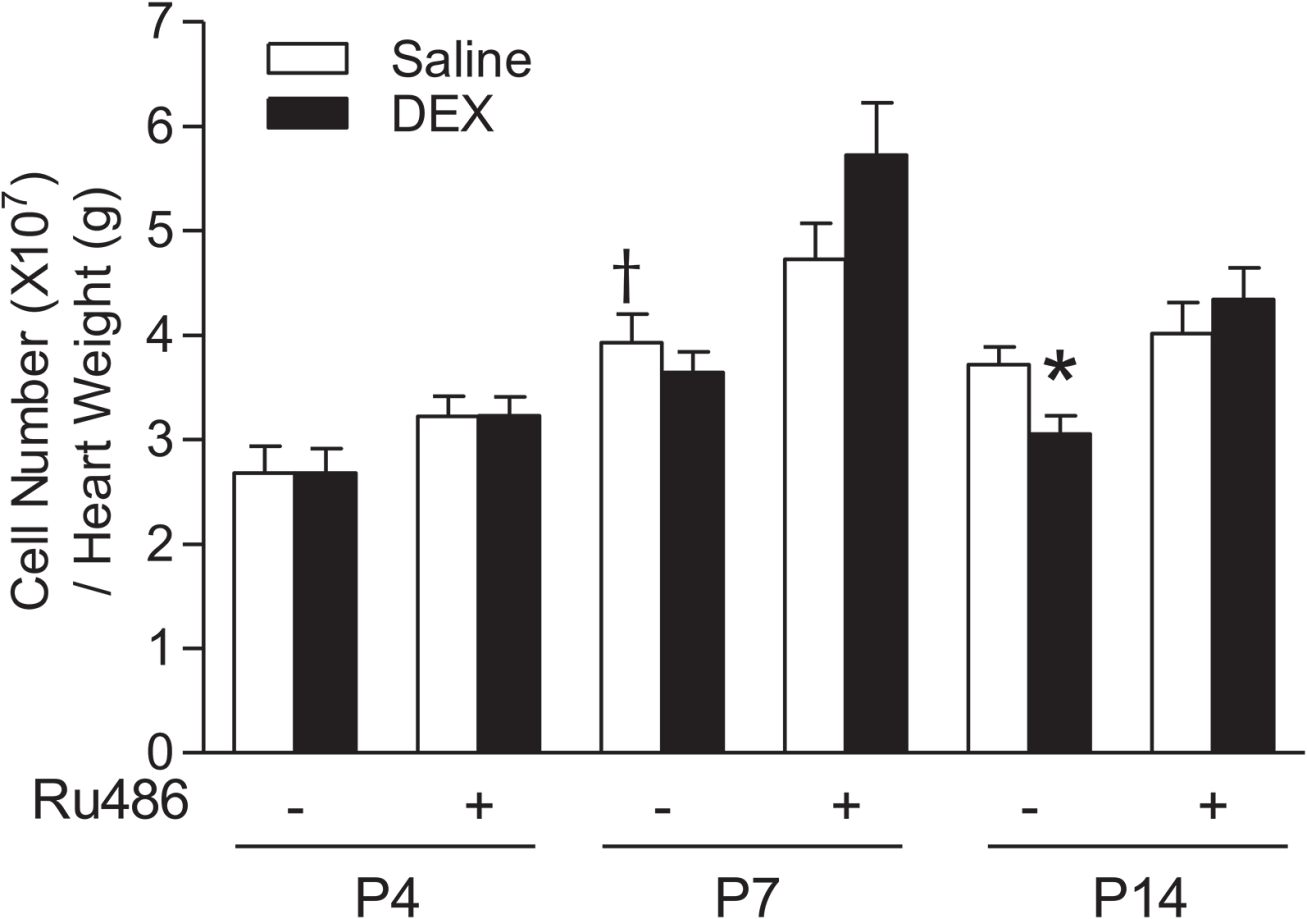
Effect of dexamethasone (DEX) on cardiomyocyte number in neonatal rats. Newborn rats were treated with tapered dose of DEX in the absence or presence of Ru486 during the first three days of postnatal life. Ru486 was administered 30 minutes prior to the DEX treatment. Cardiomyocytes isolated from day 4 (P4), day 7 (P7) and day 14 (P14) neonatal hearts were counted and normalized to per gram of heart weight. Data are mean ± SEM, n = 6–20. * p<0.05, DEX *vs*. Saline; † p<0.05, P7 *vs*. P4.

Because cardiomyocytes are largely non-proliferative in the matured heart, early endowment can potentially dictate adult cardiac function. To examine whether dexamethasone treatment influences cardiomyocyte number, we counted cardiomyocytes and normalized it to heart weights for P4, P7 and P14 animals. Fig 4 shows the effect of dexamethasone on cardiomyocyte number in the developing heart. In control animals, cardiomyocyte number per heart weight increased from P4 to P7 pups, with no further increase in P14 pups. The dexamethasone treatment had no significant effect on cardiomyocyte number in P4 and P7 pups, but significantly decreased myocyte number in the heart of P14 pups (Fig 4). This effect of dexamethasone was blocked by Ru486 (Fig 4). We also evaluated whether dexamethasone impacted cardiomyocyte size. The mononucleate and binucleate cells were scored separately with immunocytochemistry staining. Binucleated cardiomyocytes were significantly larger than mononucleate cells, as expected. During the early developmental ages of P4 and P7 pups, neither mononucleate nor binucleate cardiomyocytes increased in cell size (data not shown). In addition, neither dexamethasone nor Ru486 had significant effects on the cell size of mononucleated or binucleated cardiomyocytes (data not shown).

### 5-AZA inhibits dexamethasone-induced effects on heart development in neonatal rats

**Fig 5:**
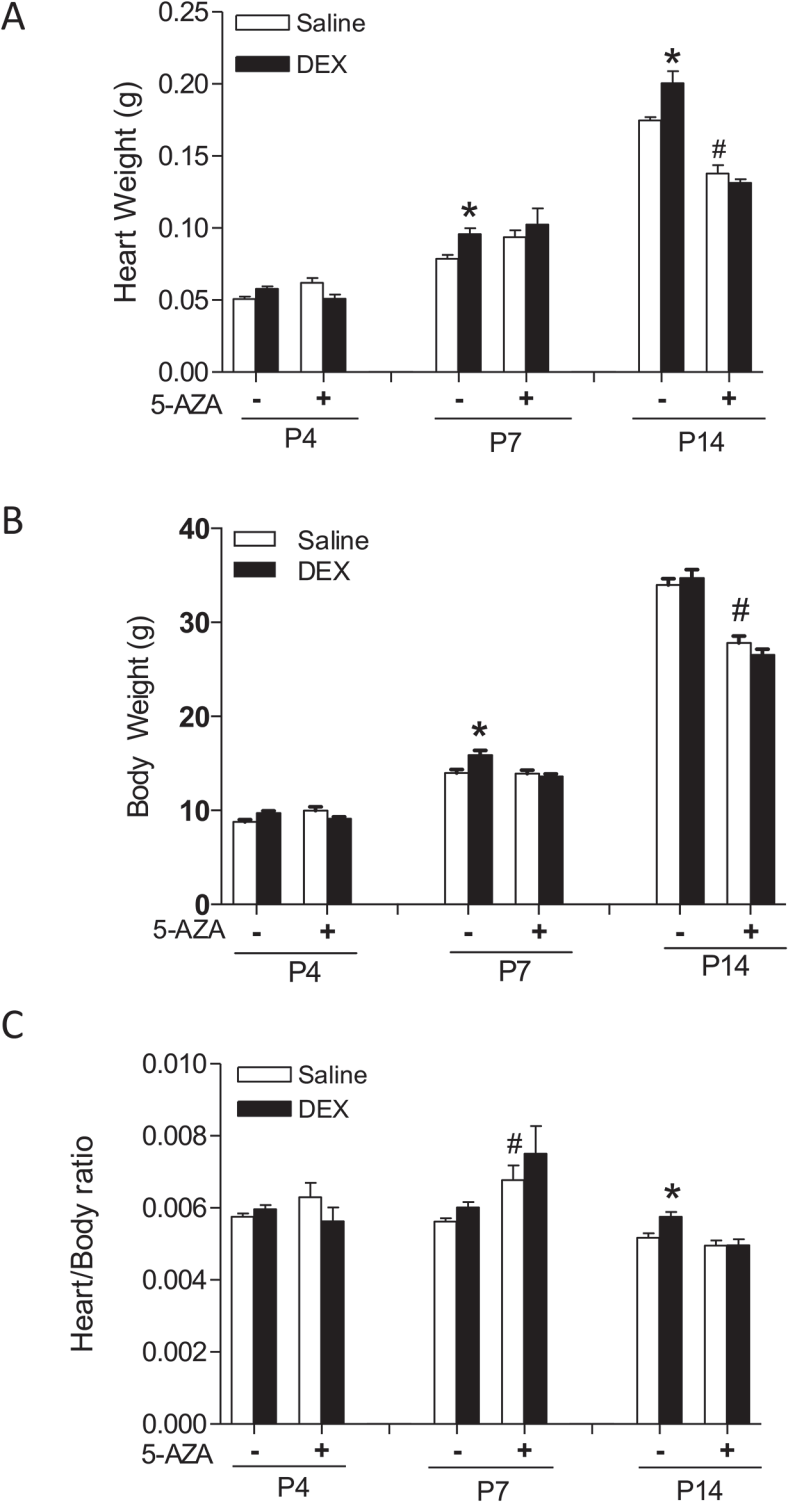
5-AZA inhibits dexamethasone (DEX)-mediated effects on heart development in neonatal rats. Newborn rats were treated with tapered dose of DEX in the absence or presence of 5-AZA during the first three days of postnatal life. 5-AZA was administered 30 minutes prior to the DEX treatment. Heart and body weights were determined in day 4 (P4), day 7 (P7) and day 14 (P14) neonatal rats. Data are mean ± SEM, n = 5–21. * p<0.05, DEX *vs*. Saline; # p<0.05, +5-AZA *vs*. -5-AZA.

Given that epigenetic modifications play an important role in the heart development and the activation of GR has been noted to alter the methylation pattern of multiple genes, we determined whether heightened methylation played a role in dexamethasone-induced effects on the heart development in neonatal rats. The effects of dexamethasone was evaluated in the absence or presence of a methylation inhibitor 5-AZA. Although 5-AZA treatment alone had no significant effects on either heart weight or body weight in P4 and P7 pups, it caused a significant but symmetric decrease in both heart and body weight in P14 pups (Figs 5A and 5B). Interestingly, although it had no significant effects on either heart or body weight in P7 pups, it significantly increased the heart to body ratio in P7 pups (Fig 5C). Of importance, in the presence of 5-AZA, effects of dexamethasone on heart weight, body weight and the heart to body weight ration were abrogated (Fig 5), suggesting DNA methylation as a critical mechanism of dexamethasone effects.

### 5-AZA blocks dexamethasone-mediated premature cardiomyocyte terminal differentiation

**Fig 6:**
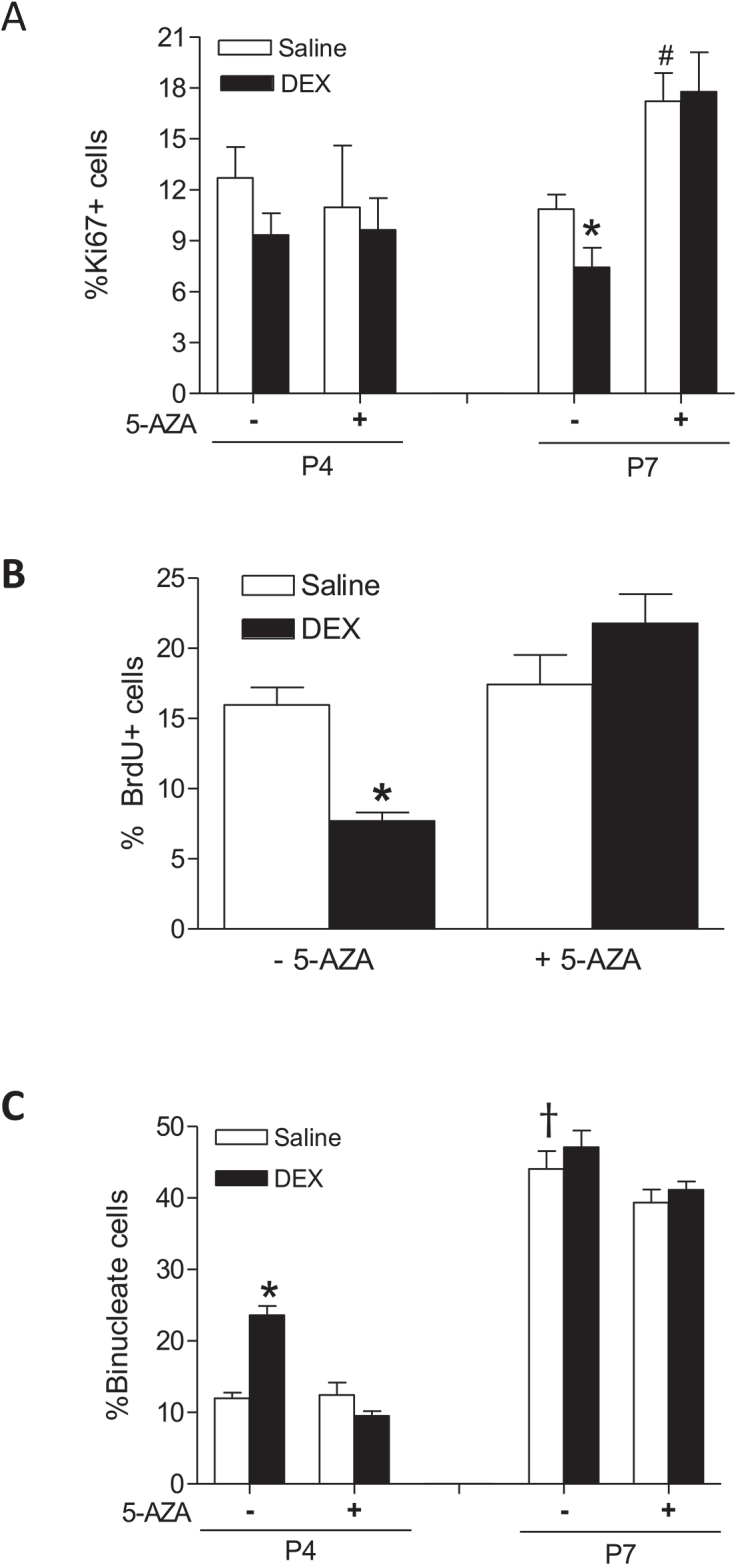
5-AZA blocks dexamethasone (DEX)-induced effects on cardiomyocyte proliferation and binucleation in neonatal rats. Newborn rats were treated with tapered dose of DEX in the absence or presence of 5-AZA during the first three days of postnatal life. 5-AZA was administered 30 minutes prior to the DEX treatment. Panel **A**: Cardiomyocytes isolated from day 4 (P4) and day 7 (P7) neonatal hearts were double stained with α-actinin and Ki67, nuclei were stained with Hoechst. Panel **B**: Cardiomyocytes isolated from P7 neonatal hearts were examined for BrdU incorporation. Panel **C**: Cardiomyocytes isolated from P4 and P7 neonatal hearts were stained with α-actinin and Hoechst, and mononucleated and binucleated cells were determined. Data are mean ± SEM, n = 4–14. * p<0.05, DEX *vs*. Saline; # p<0.05, +5-AZA *vs*. -5-AZA; † p<0.05, P7 *vs*. P4.

The effects of 5-AZA on dexamethasone-induced premature cardiomyocyte binucleation and inhibition of proliferation were further studied. As shown in Fig 6A, the dexamethasone-induced decrease of Ki67-positive cardiomyocytes in P7 pups was blocked by 5-AZA. Similarly, dexamethasone-mediated suppression of BrdU incorporation was inhibited by 5-AZA (Fig 6B), suggesting a methylation-dependent mechanism in dexamethasone-induced decease in cardiomyoctye proliferation. Of interest, 5-AZA alone significantly increased Ki67-positive cardiomyocytes, despite the effects of dexamethasone (Fig 6A). Although 5-AZA alone had no significant effect on cardiomyocyte binucleation, it inhibited the dexamethasone-induced increase of percent binucleated cells in the hearts of P4 pups (Fig 6C).

### 5-AZA abrogates the effects of dexamethasone on cardiomyocyte endowment

**Fig 7:**
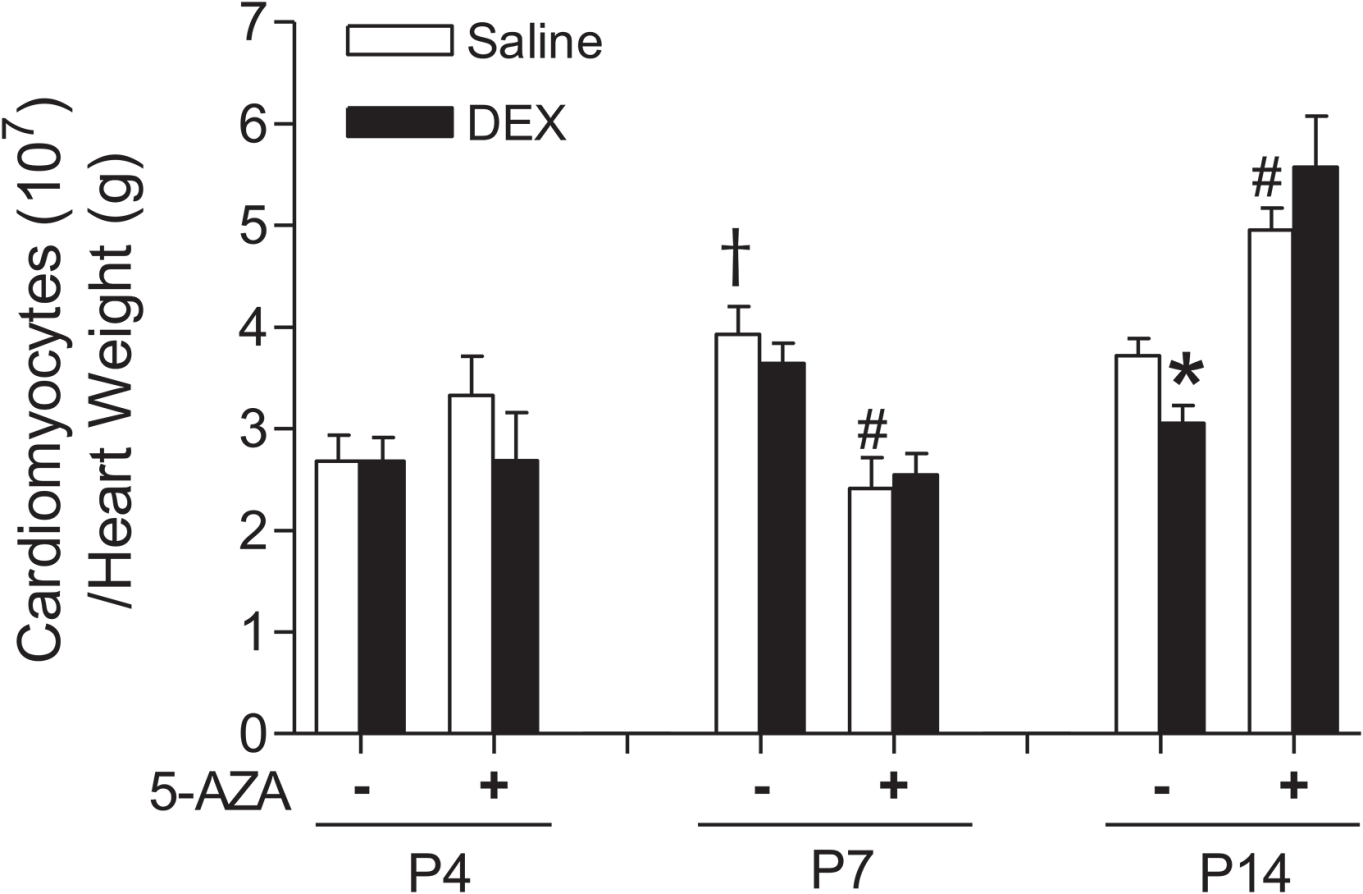
5-AZA abrogates dexamethasone (DEX)-mediated effects on cardiomyocyte number in neonatal rats. Newborn rats were treated with tapered dose of DEX in the absence or presence of 5-AZA during the first three days of postnatal life. 5-AZA was administered 30 minutes prior to the DEX treatment. Cardiomyocytes isolated from day 4 (P4), day 7 (P7) and day 14 (P14) neonatal hearts were counted and normalized to per gram of heart weight. Data are mean ± SEM, n = 5–20. * p<0.05, DEX *vs*. Saline; # p<0.05, +5-AZA *vs*. -5-AZA; † p<0.05, P7 *vs*. P4.

As shown in Fig 7, in P4 pups, 5-AZA treatment did not influence cardiomyocyte numbers in either saline or dexamethasone group. In P7 pups, a significant reduction of cardiomyocyte number was induced by 5-AZA alone. In contrast, in P14 pups, 5-AZA treatment resulted in a significant increase of cardiomyocyte number in both saline and dexamethasone groups. Of importance, the dexamethasone-induced decrease of cardiomyocyte number in P14 pups was abrogated by 5-AZA (Fig 7). These data provide novel evidence of developmental specific effect of DNA methylation on cardiomyocyte number in the critical window of the heart development. The results may be interpreted as evidence for a role of DNA methylation in regulating cardiomyocyte proliferation. Inhibition of methylation may potentially affect key regulatory proteins essential to proliferation in the developing heart.

### 5-AZA inhibits dexamethasone-induced down-regulation of cyclin D2 in the heart

**Fig 8:**
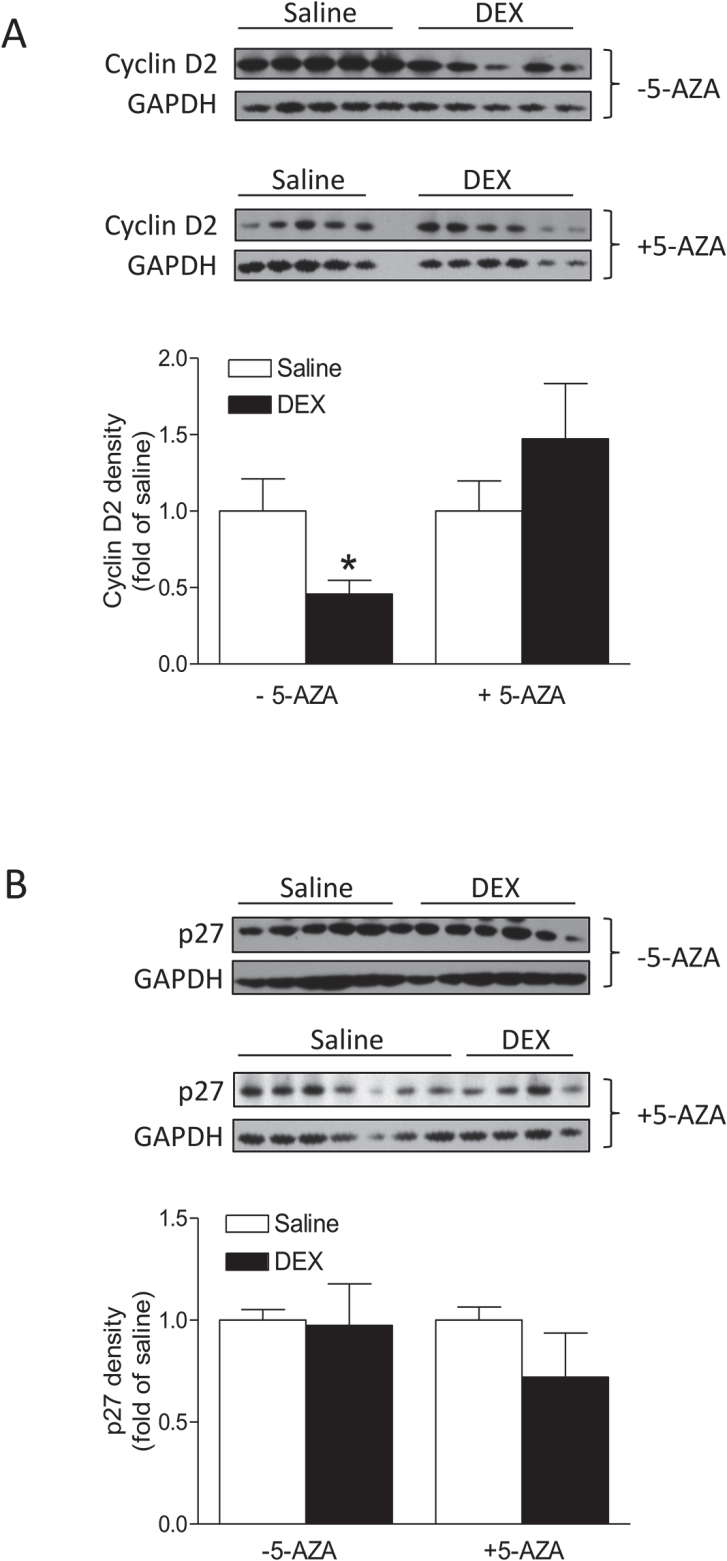
5-AZA blocks dexamethasone (DEX)-induced down-regulation of cyclin D2 in the heart. Newborn rats were treated with tapered dose of dexamethasone (DEX) in the absence or presence of 5-AZA during the first three days of postnatal life. 5-AZA was administered 30 minutes prior to the DEX treatment. Protein was isolated from day 4 (P4) neonatal hearts and protein abundance of cyclin D2 (**A**) and p27 (**B**) was determined by Western blot. Data are mean ± SEM, n = 5–6 * p<0.05, DEX *vs*. Saline.

To examine potential target genes involved in dexamethasone-mediated regulation of the proliferation and binucleation of cardiomyocyte, we determined the protein abundance of cyclin D2 and p27 in the hearts of P4 pups, which play an important role in the regulation of cell cycle activity. As shown in Fig 8A, the dexamethasone treatment significantly decreased cyclin D2 expression in the heart, which was blocked by 5-AZA. In contrast, dexamethasone had no significant effect on p27 expression either in absence or the presence of 5-AZA (Fig 8B).

### Dexamethasone does not change global methylation of the heart

**Fig 9:**
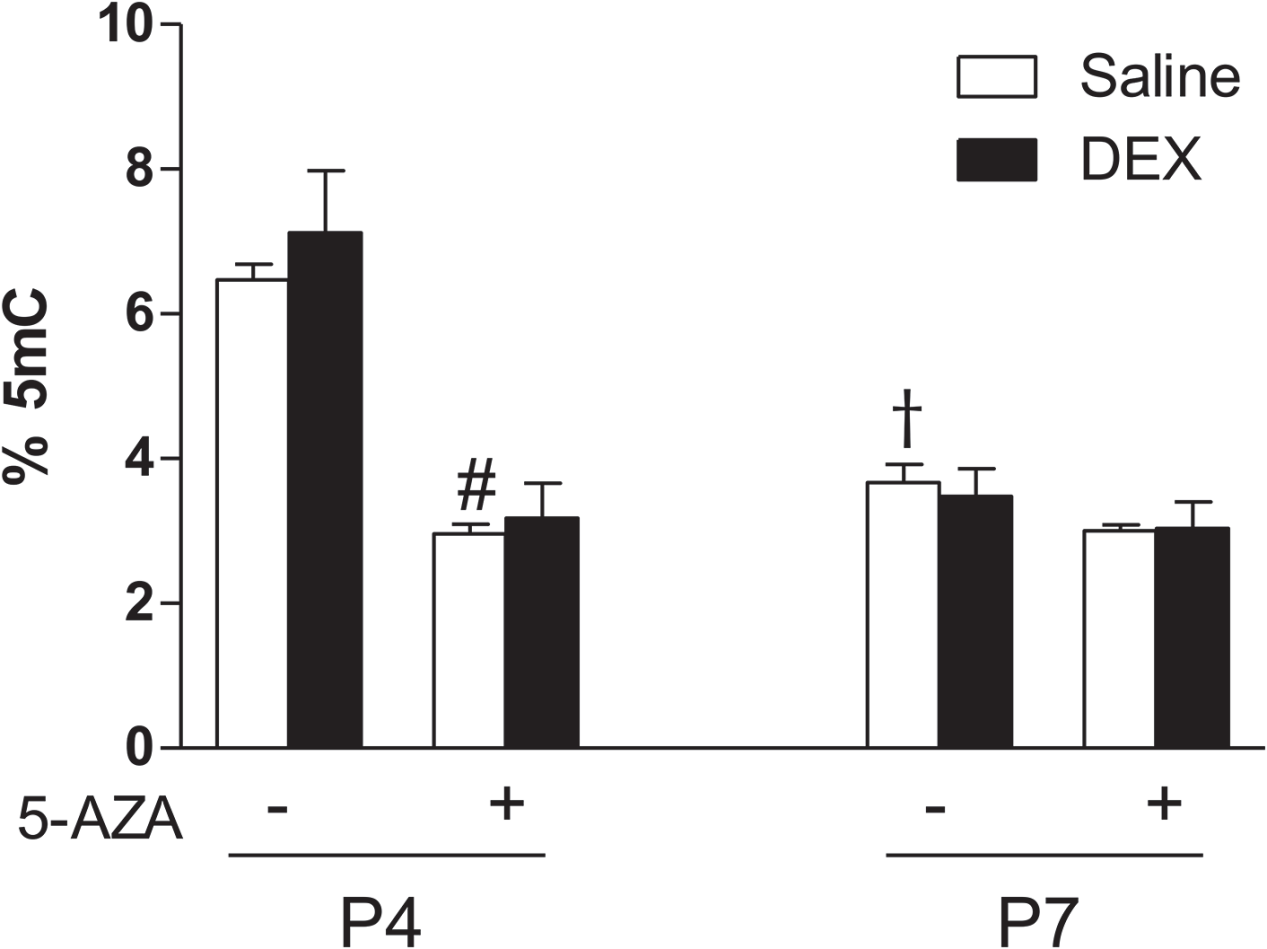
5-AZA decreases DNA methylation levels in neonatal hearts. Newborn rats were treated with tapered dose of dexamethasone (DEX) in the absence or presence of 5-AZA during the first three days of postnatal life. 5-AZA was administered 30 minutes prior to the DEX treatment. Genomic DNA was extracted from day 4 (P4) and day 7 (P7) neonatal hearts, and methylation levels were measured using a 5-mC ELISA kit. Data are mean ± SEM, n = 5–6. # p<0.05, +5-AZA *vs*. -5-AZA; † p<0.05, P7 *vs*. P4.

Genomic DNA was extracted from the whole hearts, and a 5-methyl cytosine detection kit was used to analyze global methylation levels of DNA from each group. In saline control animals, there was a development-dependent decrease in global methylation in the heart, and DNA methylation levels were significantly reduced in P7 pup hearts as compared with P4 pups (Fig 9). In P4 pups, 5-AZA significantly decreased the DNA methylation level in the heart, albeit the dexamethasone treatment had no significant effect on the level of methylated 5-methyl cytosine (Fig 9). In P7 pups with reduced DNA methylation levels in the heart, 5-AZA had no further effect on the methylation level, regardless of saline or dexamethasone treatments (Fig 9).

## Discussion

The synthetic glucocorticoid dexamethasone is commonly used to reduce the morbidity of respiratory complications in preterm infants. Yet, the potential adverse effects of dexamethasone therapy on the developing heart remain unknown. In the present study, we examined the impact of clinically relevant neonatal doses of dexamethasone on cardiomyocyte proliferation and binucleation in the developing heart. The results provided evidence of glucocorticoid-mediated stimulation of premature cardiomyocyte binucleation, inhibition of myocyte proliferation, and reduction in total cardiomyocyte number during the critical window of the heart development. We demonstrated that the dexamethasone-induced effects were abrogated by a GR antagonist Ru486, and thus revealed the GR-mediated effect on premature heart development in newborns. In addition, we provide novel evidence of a potential mechanism of DNA methylation in GR-mediated effects in the developing heart. The results provided insights in the regulation of cardiomyocyte maturation by endogenous glucocorticoids and the underlying mechanisms that may be involved.

Dexamethasone has been widely used in clinic to prevent the morbidity of chronic lung disease in preterm infants. In addition to its effect on the lung, glucocorticoids are also essential regulators of the development of other organs such as the brain and heart [20,21]. Given that the developmental stage of hearts and brains in newborn rats is somewhat equivalent to that of the fetal development in the third trimester of human gestation, they provide a model in studying the effect of dexamethasone therapy in preterm infants on the heart and brain development. Recently, Chang and colleagues uncovered that neonatal dexamethasone treatment altered the susceptibility of the immature brain to hypoxic-ischemic brain injury [21]. Studies also have provided evidence for negative occurrences with the dexamethasone treatment including myocardial hypertrophy [22] and premature death [5]. De Vries and colleagues investigated the long-term effect of neonatal dexamethasone therapy on cardiac function, and showed that the heart to body weight ratio was increased in P7 pups but decreased in 45-week-old rats [23]. Similar to this study, our results also showed an increase in the heart to body weight ratio in P14 pups.

It has been reported that dexamethasone treatment increased cardiomyocyte length, width and volume at 45 week [23,24], suggesting neonatal dexamethasone therapy had a long-term effect on the heart and may be responsible for the cardiomyocyte hypertrophy later in life in adulthood. However, the underlying mechanisms at the cellular and molecular levels remain elusive. The present study provided a mechanism in the understanding of this long-term effect of neonatal dexamethasone therapy, and demonstrated that dexamethasone treatment significantly suppressed proliferation and promoted premature maturation of cardiomyocytes, resulting in a decreased total cardiomyocyte number in the developing heart. The finding that dexamethasone-mediated stimulation of premature cardiomyocyte binucleation and inhibition of myocyte proliferation were blocked by Ru486 demonstrates the GR-mediated effects during this critical window of the heart development.

In the developing heart, cardiomyocytes are initially mononucleate and proliferative, but proliferation capacity is soon limited as mature cardiomyocytes undergo binucleation and hence are non-proliferative [10,11]. Fetal exposure to glucocorticoids has been shown to stimulate binucleation in preterm piglets [25], but has no overall effects in fetal lambs [26]. Because rodent cardiomyocyte terminal differentiation starts and continues within the first two weeks of postnatal life, which is equivalent to human heart development in late fetal stage of third trimester, neonatal rats provide a reasonable animal model to study the effect of glucocorticoid treatment on preterm infants at the critical window of the heart development. The present study demonstrates that dexamethasone treatment of newborn rats stimulates premature cardiomyocyte binucleation in day 4 pups. The transition to binucleation is a critical time in the heart development because of the limited proliferative capacity thereafter. Therefore, premature binucleation can potentially depress cardiomyocyte endowment. Since cardiomyocytes are the functional unit of the heart, adequate numbers are essential for cardiac function in adaption to physiological changes. Indeed, our results showed decreased cardiomyocyte proliferation on P4 and P7 pups, as well as a significantly lower cardiomyocyte number in P14 pups. Since the majority of cardiomyocytes are binucleated and terminally differentiated at this developmental age, it is likely that the dexamethasone-induced reduction of cardiomyocyte number will persist into adulthood and contribute to heart dysfunction later in life. The depressed cardiomyocyte number was also reported with other stressor such as intrauterine growth restriction [27]. This raises a major concern, when cardiomyocyte numbers are lowered in the heart, the remaining cardiomyocytes may undergo cellular hypertrophy to generate sufficient contractile forces. This compensation is not without consequence in increased risk of ischemic heart disease. Although the present study focuses on the effect of glucocorticoid on the critical window of the heart development in cardiomyocyte maturation and binucleation, which is of clinical relevance in the glucocorticoid therapy in preterm infants, the potential effect of glucocorticoid on the heart development in the earlier embryonic stage remains an interesting question for further investigation.

Heart growth occurs by one of two methods, hyperplasia or hypertrophy, and fetal glucocorticoid exposure was noted to induce both hyperplasic [26,28] and hypertrophic growth [29]. In neonates, complicated and sometimes contradictory accounts have also been noted. Studies have revealed that the glucocorticoid treatment is associated with hyperplasic [30], hypertrophic [31,32] or no effect on the growth [33] of cardiomyocytes, as analyzed by the protein to DNA ratio. Formation of binucleated myocardial cells is regarded as an early indicator of transition of hyperplasia to hypertrophic growth [10], indicating ceasing of proliferation of cardiomyocytes. Mounting evidence has shown that arrest of the cell cycle, and thereby locking of cells in either G1 or G0 phase stop myocyte proliferation [34,35]. Poolman and colleagues studied the profile of the cell cycle in cardiomyocytes in postnatal rats from P2 to P5, and found that the percentage of G0/G1 phase cells was increased during the early neonatal development [36]. These studies suggested that cell cycle dependent molecules, such as cyclins, cyclin-dependent kinase (CDK) and CDK inhibitors (CDI), may control the transition from hyperplasia to hypertrophic growth of myocytes [37,38]. Indeed, CDI p21 was up-regulated during the switch of cardiac myocyte from hyperplasia to hypertrophic growth in rats [37]. G1 phase acting proteins, CDK4 and CDK6 were also up-regulated in this process and were associated with hypertrophic growth of myocytes [36,39].

It is possible that increased exposure to glucocorticoids during the critical window of the heart development may alter the expression or activities of these key regulating proteins of the cell cycle. The primary mechanism of glucocorticoid activity is *via* the GR, an intracellular ligand dependent transcription factor. The GR plays a critical role in fetal heart maturation and cardiovascular health and disease. Knockout of the GR gene in cardiomyocytes results in impaired cardiac structure and function that can be observed at embryonic day 17.5 [40], and the offspring die prematurely from spontaneous cardiovascular disease [41]. In glucocorticoid null fetal mice hyper-proliferation was reported, implicating the GR as an important regulator of cell proliferation [42]. Several lines of evidence suggest the role of glucocorticoids in regulating the cell cycle. It has been shown that dexamethasone suppresses rat epithelial cell growth by blocking a specific cell cycle of either G1 or G0 phase, and dexamethasone withdrawal increases the expression of G1 marker genes, including c-Myc and cyclin D1 [43]. In addition, glucocorticoid signaling arrests cell cycle activity by either transcriptional repression of G1 phase kinases CDK4/6 or enhanced transcription of CDK endogenous inhibitor, CDIs p21 and p27 [42]. Moreover, a recent study has shown that dexamethasone up-regulates CDI p21 and inhibits osteoblastic cell proliferation and these effects are blocked by the GR antagonist Ru486 or specific silencing of GR, demonstrating a GR-dependent mechanism [44]. Further studies are needed to investigate the effect of dexamethasone on the cell cycle regulating molecules in cardiomyocytes of the development heart.

Although the mechanisms by which perinatal dexamethasone treatment causes long-term effects are still not known, increasing evidence indicates that epigenetic modifications, such as DNA methylation, play an important role. Accumulating evidence indicates interaction between GR and proteins involved in methylation, such as DNMT3b and MeCP2, leading to promoter methylation of genes [45]. In a recent study, Crudo and colleagues found that prenatal exposure to synthetic glucocorticoids resulted in an altered global DNA methylation pattern in an organ and development dependent manner, and these changes were also observed in the next generation, indicating dexamethasone-caused methylation as a long-term manipulation in the gene regulation [17]. Many mammalian genes possesses glucocorticoid-response element (GRE), and the expression is subjected to regulation by GR activation. GR binding to targets genes leads to change of methylation status on the gene promoters [18,46]. In the present study, inhibition of DNA methylation by 5-AZA blocked the dexamethasone-induced changes in cardiomyocyte proliferation and reversed the dexamethasone-induced decrease of cardiomyocyte number, providing novel evidence of DNA methylation in dexamethasone-induced change of gene regulation and heart development. Of importance, these findings suggest a potential strategy to abrogate or reverse the dexamethasone-induced adverse effects, considering the routine administration of dexamethasone in clinical practice to deal with preterm risks. Despite the effect of dexamethasone, the finding that 5-AZA alone promoted cardiomyocyte proliferation at P7 pups resulting in a significant increase in cardiomyocyte number in P14 hearts is intriguing and suggests an important role of DNA methylation in the heart development. This is in agreement with the findings in a study by Kou *et al*., which showed that DNA synthesis was increased in cardiomyocytes treated with 5-AZA [47].

Evidence from recent studies indicates that DNA methylation may be an important mechanism in cell cycle regulation. In Hela cells, multiple DNA sequences are found differentially methylated between G0 and S phase, suggesting dynamic methylation is involved in control of cell cycle [48]. Indeed, cell cycle regulating proteins are targets of epigenetic regulation and one example is the CDI protein P16, which inhibits CDK4/CDK6 activity and leads to G1-cell cycle arrest. Extensive methylation of the p16 promoter results in inactivation of P16 and cell proliferation [49]. In the present study, we found that the dexamethasone treatment had no significant effect on global methylation levels in the hearts of P4 and P7 pups. Given the finding that inhibition of methylation by 5-AZA abrogated dexamethasone-induced effects on cardiomyocyte proliferation and binucleation, it is possible that instead of a genome-wide effect, GR-mediated methylation may be gene specific in the developing heart. It has been shown that dexamethasone has differential effects in the regulation of methylation that is organ-dependent and gene-dependent in an organ [17,18]. Indeed, the present study demonstrated that dexamethasone significantly decreased cyclin D2 (a cell cycle promoter), but not p27 (a cell cycle inhibitor), in the heart of P4 pups, which was blocked by 5-AZA. This suggests that methylation-dependent down-regulation of cyclin D2 may play a role in dexamethasone-induced decrease in cardiomyocyte proliferation. The finding of a development-dependent decrease in global methylation from P4 to P7 hearts is intriguing given that a study conducted by Kou and colleagues reported that global methylation increased progressively with age in cardiomyocytes [47]. A possible reason of this inconsistency between the two studies may be the difference in sample source. In the present study we isolated DNA from the whole heart that is composed of many cell types including fibroblasts, endothelial cells, smooth muscle cells and cardiomyocytes, whereas Kou *et al*. analyzed global DNA methylation in isolated and cultured cardiomyocytes [47]. Nonetheless, alteration of methylation during development or aging has been noted in a variety of organs, including the heart [50,51].

The present study provides novel evidence of dexamethasone-mediated premature terminal differentiation of cardiomyocytes at the critical window of heart development during early postnatal life. Dexamethasone promotes premature exit of the cell cycle and cardiomyocyte binucleation, leading to a significant decrease of proliferating cells. These effects result in a decrease in cardiomyocyte endowment in the heart. Although the present finding suggests an important role of DNA methylation in dexamethasone-mediated regulation of cardiomyocyte proliferation and binucleation in the developing heart, other mechanisms, e.g. histone modifications may not be excluded. Whereas protein methylation and DNA methylation are mediated by different mechanisms, it remains to be explored whether histone methylation is also involved in the dexamethasone-induced effects.
